# In-Stent Restenosis Overview: From Intravascular Imaging to Optimal Percutaneous Coronary Intervention Management

**DOI:** 10.3390/medicina60040549

**Published:** 2024-03-28

**Authors:** Neda Shafiabadi Hassani, Lucas Carlini Ogliari, Pedro Rafael Vieira de Oliveira Salerno, Gabriel Tensol Rodrigues Pereira, Marcelo Harada Ribeiro, Luis Augusto Palma Dallan

**Affiliations:** 1 Harrington Heart and Vascular Institute, University Hospitals Cleveland Medical Center, Cleveland, OH 44106, USA; neda.shafiabadihassani@uhhospitals.org (N.S.H.); pedrorafael.salerno@uhhospitals.org (P.R.V.d.O.S.); gabriel.tensolrodriguespereira@uhhospitals.org (G.T.R.P.); 2 Intravascular Imaging Core Laboratory, University Hospitals Cleveland Medical Center, Cleveland, OH 44106, USA; 3 SOS Cardio Hospital and Imperial Hospital de Caridade, Florianópolis 88020-210, SC, Brazil; lcogliari@gmail.com (L.C.O.); mhr60@msn.com (M.H.R.)

**Keywords:** in-stent restenosis, intravascular imaging, percutaneous coronary intervention management

## Abstract

Despite ongoing progress in stent technology and deployment techniques, in-stent restenosis (ISR) still remains a major issue following percutaneous coronary intervention (PCI) and accounts for 10.6% of all interventions in the United States. With the continuous rise in ISR risk factors such as obesity and diabetes, along with an increase in the treatment of complex lesions with high-risk percutaneous coronary intervention (CHIP), a substantial growth in ISR burden is expected. This review aims to provide insight into the mechanisms, classification, and management of ISR, with a focus on exploring innovative approaches to tackle this complication comprehensively, along with a special section addressing the approach to complex calcified lesions.

## 1. Introduction

Stents can be classified based on various factors such as composition, design, and drug-elution properties under two main bare-metal (BMS) and drug-eluting (DES) types. Understanding these classifications is crucial for selecting the most appropriate stent type for individual patients and mitigating the risk of complications. The most common indications for stent implantation include the treatment of coronary artery disease, acute coronary syndromes, and symptomatic angina. Percutaneous coronary intervention (PCI) has undergone a transformative evolution spanning four decades, transitioning from balloon angioplasty and bare-metal stents (BMSs) to the current drug-eluting stent (DES) era [1]. Despite significant advances in improving outcomes, there are still persistent challenges arising from stent technology. The stent struts, polymers, and drugs eluted from the stents may lead to vascular injury, which serves as the foundation for processes such as fibroblast proliferation, neointimal hyperplasia, and, ultimately, in-stent restenosis (ISR) [2]. By combining the metallic stent platform with a polymer releasing an antiproliferative drug, DES significantly improved the efficacy of PCI by suppressing the formation of neointimal hyperplasia (NIH) and reducing the risk of ISR. While the proportion of patients with BMS-ISR has been substantially reduced with the introduction of DES technology, ISR is still encountered in about 5 to 15% of PCIs in the United States [3,4]. Still, in the DES era, the overall prevalence of PCI performed in the United States due to clinical ISR remained relatively unchanged over the years, accounting for about one out of ten interventions and, therefore, remaining a significant problem even to this day [5].

ISR risk factors, such as obesity and diabetes, continue to increase, and technological advancements have allowed the treatment of progressively more complex lesions using PCI, which are inherently more prone to ISR; therefore, the number of ISR-PCIs is expected to increase [6]. Moreover, clinical ISR may be currently underdiagnosed, as it is identified at a lower rate when compared to imaging or physiology findings [7]. Given the substantial number of stent deployments globally, ISR has evolved into a pathology with an increasing socioeconomic burden, ultimately leading to higher healthcare costs [2]. The growing recognition of ISR as a public health concern mandates a more comprehensive and holistic approach to both its evaluation and treatment.

This review aims to provide insight into the mechanisms, classification, and management of ISR using newly proposed approaches for medical and interventional treatment. The focus is on exploring innovative approaches to tackle this issue comprehensively, along with a special section addressing the approach to complex calcified lesions.

## 2. In-Stent Restenosis

### 2.1. Definition

**Figure 1:**
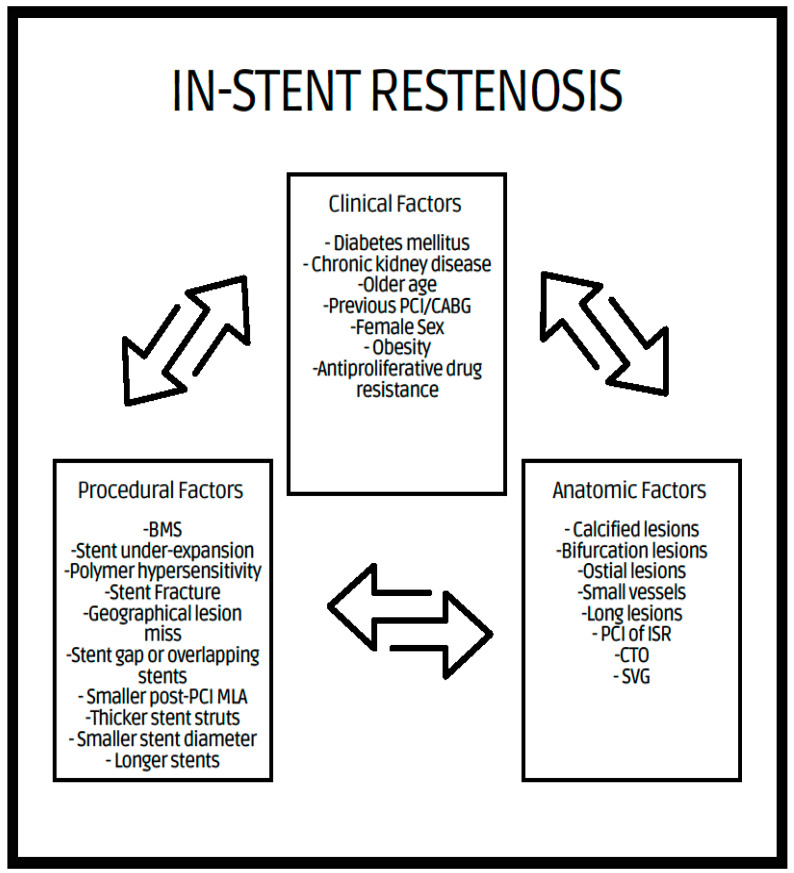
Clinical, procedural, and anatomical factors associated with ISR. BMS = bare-metal stent, PCI = percutaneous coronary intervention, MLA = minimum lumen area, CABG = coronary artery bypass graft, CTO = chronic total occlusion, SVG = saphenous vein graft.

In-stent restenosis (ISR) is defined as luminal renarrowing of greater than 50% within 5 mm of a stent edge on follow-up angiography [8,9,10]. Clinical restenosis occurs with (1) luminal renarrowing of greater than 50% of the minimal luminal diameter (MLD) associated with either symptoms of ischemia or abnormal results of invasive diagnostic testing such as fractional flow reserve (FFR) (<0.80) or intravascular imaging (<6 mm for left main or <4 mm for non-left main) or (2) luminal renarrowing of greater than 70% even in the absence of ischemic signs or symptoms [11]. Clinical restenosis typically leads to repeat target lesion revascularization (TLR), either through percutaneous coronary intervention (PCI) or coronary artery bypass surgery (CABG). Clinical, procedural, and anatomic factors have key roles in the pathogenesis of ISR, as described by Giustino et al., and are shown in Figure 1 [2].

### 2.2. Mechanism of ISR

Compared to BMS-ISR, DES-ISR has decreased neointimal proliferation. DESs minimize neointimal proliferation through localized delivery of antiproliferative drugs with programmed pharmacokinetics, thus controlling smooth muscle cell growth and migration as well as preventing inflammatory responses. In contrast, hypersensitivity to the polymer and the drug, local inflammation, and delayed healing are the main contributors to neointima formation with DES-ISR [12].

There are several differences between BMS-ISR and DES-ISR. BMS-ISR tends to have an earlier presentation (around 6 months post-stent deployment) and displays a diffuse pattern of neointimal proliferation composed of vascular smooth muscle cells and extracellular matrix [12]. In contrast, DES coating delays the intimal proliferation for several years and is often accompanied by a focal pattern involving stent edges, described as proteoglycan-rich and with less cellularity [13,14]. Furthermore, the identification of layered signal tissue echogenicity is more commonly found with DES-ISR [11].

Neoatherosclerosis, also known as in-stent new atherosclerosis, is defined by an accumulation of lipid-laden foamy macrophages sometimes accopmpanied by a necrotic core and/or calcification in the newly growing intima after stent deployment [15].

Intravascular ultrasound (IVUS) is limited in its capability to evaluate neoatherosclerosis, and optical coherence tomography (OCT) is the gold standard to diagnose this condition. OCT studies have provided valuable insights to differentiate between early ISR with homogeneous neointimal hyperplasia and late ISR, characterized by neoatherosclerosis with thin-cap fibroatheroma and lipid-rich neointima [15]. Given the predominant use of DES stent deployment in recent years, neoatherosclerosis is a potential mechanism of late stent failure [16]. Neoatherosclerosis tends to occur faster than native vessel atherosclerosis but, ultimately, may lead to the same dire consequences [17,18].

### 2.3. Intravascular Imaging

As various biological and mechanical mechanisms may subscribe to DES-ISR, the management of such daring problems seeks the identification of any underlying mechanical problems that can be hulled. IVUS and OCT allow this systematic investigation to plan interventions, face the underlying cause, and optimize the results of any necessary intervention. IVUS is a non-invasive technique for cross-sectional tissue imaging and comprises.of two basics strategies a mechanically single rotating transducer, or a solid-state or phase array design using activation of multiple transducers placed near the tip of a catheter. OCT utilises near-infrared light to achieve a tissue penetration depth of several hundred microns.

**Figure 2:**
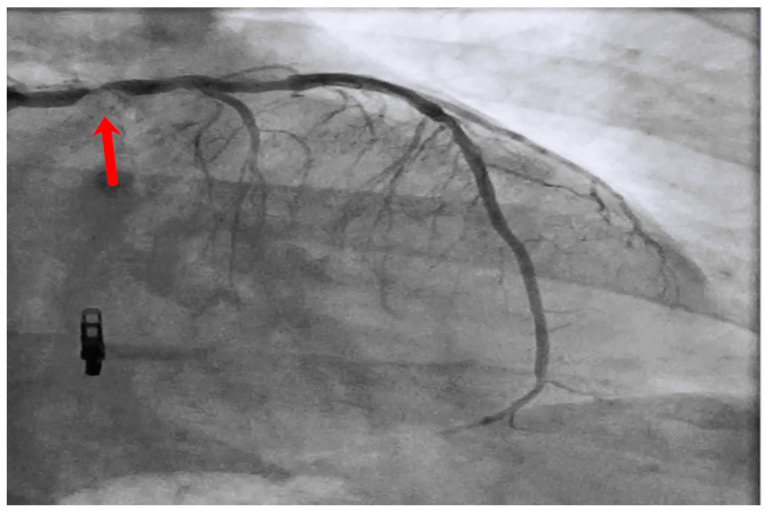
Left main stem (red arrow) in-stent restenosis.

**Figure 3:**
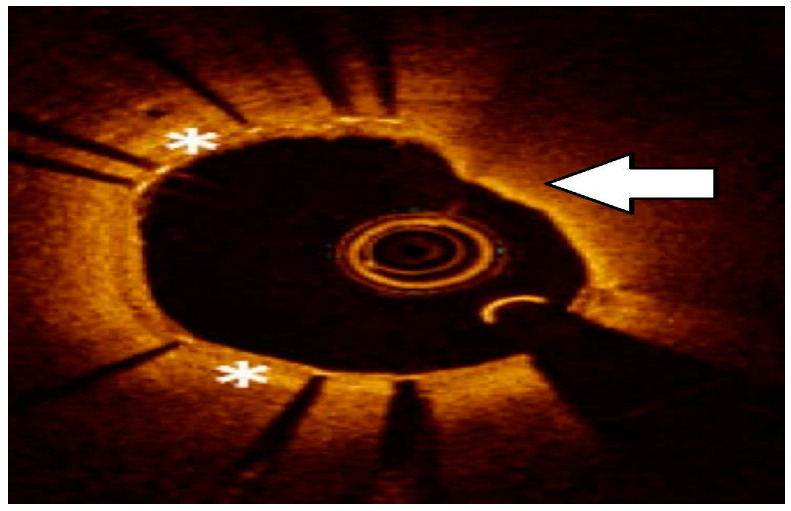
Neoatherosclerosis: heterogeneous composition with in-stent necrotic core with thin fibrous cap, lipid, or calcification and foamy macrophage accumulation. Asterisks represents Neoatherosclerosis, White arrow represents necrotic core.

**Figure 4:**
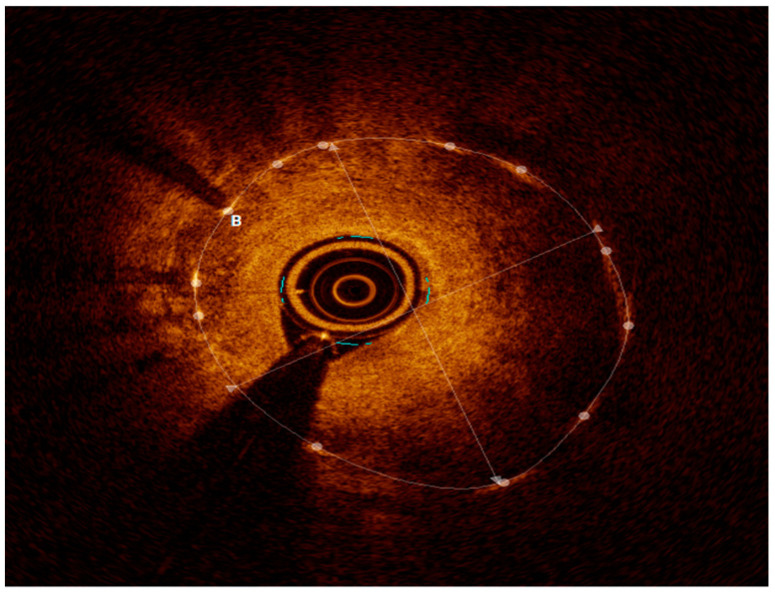
OCT with severe neointimal hyperplasia—homogeneous, bright, uniform layer, B represents neointimal hyperplasia.

**Figure 5:**
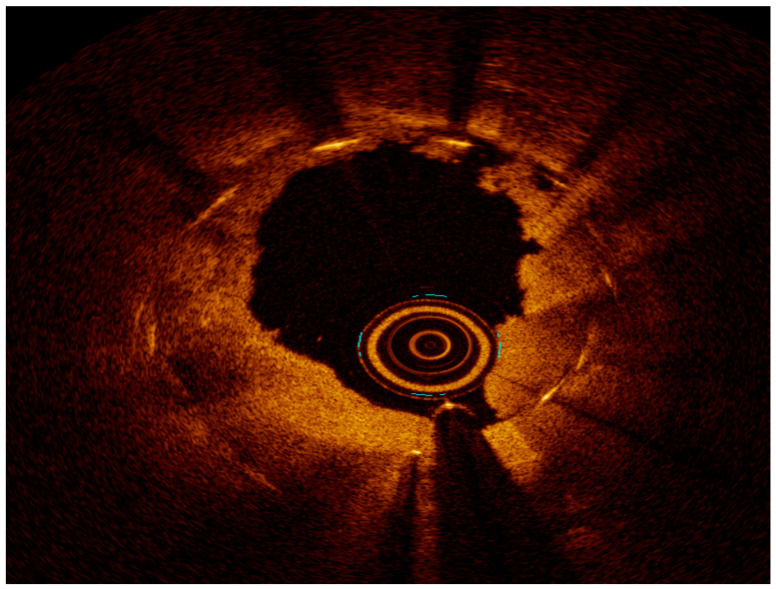
OCT after ELCA.

**Figure 6:**
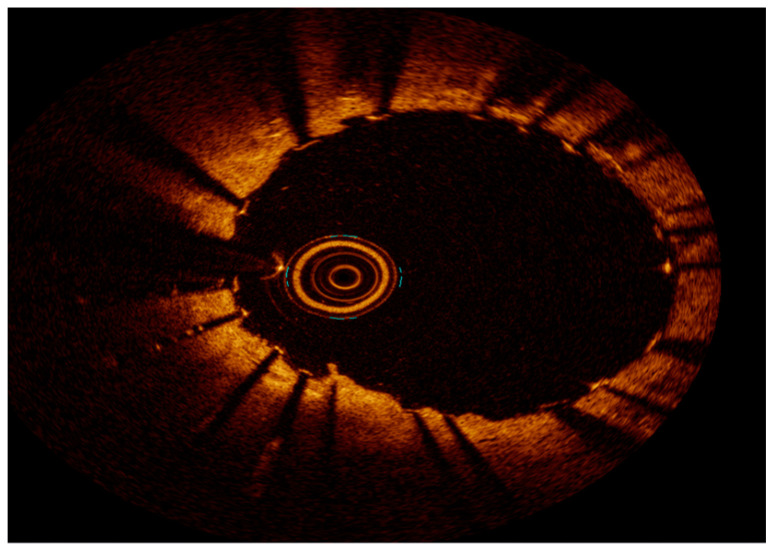
OCT final result after ELCA and DES PCI—note two stent layers.

**Figure 7:**
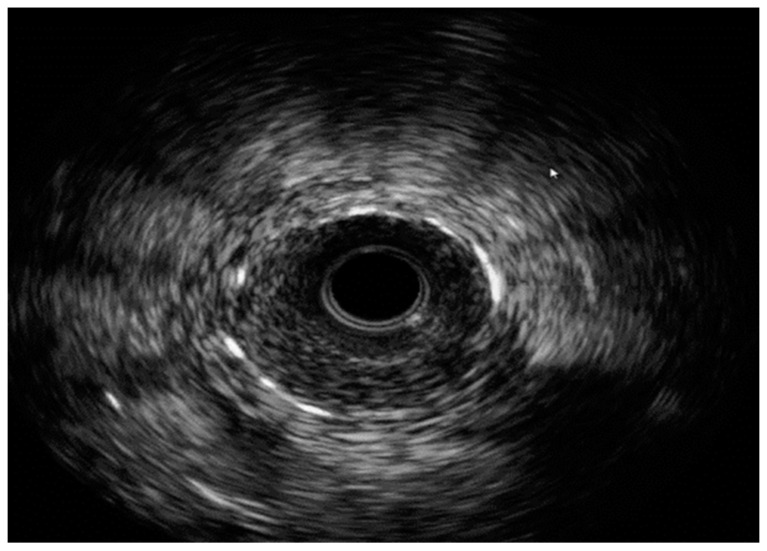
IVUS with severe ISR. Images are from author’s collection.

Intravascular imaging may provide a better understanding of the underlying ISR mechanism, helping to differentiate between mechanical (i.e., stent underexpansion or fracture) and biological causes [19,20] (Figure 2, Figure 3, Figure 4, Figure 5, Figure 6 and Figure 7). Mechanical causes of ISR may be associated with stent underexpansion, malposition, or stent fracture. Inadequate preparation in calcified lesions can lead to stent underexpansion and malapposition. In these inadequately prepared calcified lesions, deploying a stent may, in turn, hinder future interventional attempts to address suboptimal resultsFor this reason, identification and characterization of severe coronary artery calcification are essential before stent deployment [19,20]. Intracoronary imaging can, likewise, detect the number of stent layers at the lesion spot and assess the expansion of each stent layer. Thus, there is strong evidence supporting the use of intravascular imaging to determine the ISR mechanism and guide the best treatment strategy [21].

Based on current evidence, the use of IVUS or OCT has appeared to be crucial for determining the mechanism of stent failure in ISR. However, guideline recommendations for the use of intracoronary imaging are still limited. Regarding procedural optimization, the 2018 ESC/EACTS Guidelines recommend the use of IVUS for unprotected left main lesions and IVUS or OCT in selected cases (Class IIa recommendation, level of evidence B). In addition, the use of OCT and/or IVUS for the detection of stent-related mechanical issues leading to restenosis has a Class IIa indication, level of evidence C [22]. The 2021 ACC/AHA/SCAI Guidelines for Coronary Artery Revascularization use of IVUS for procedural optimization of left main or complex coronary stenting is considered Class IIa, level of evidence B-R. OCT is considered a reasonable substitute for IVUS in coronary stent implantation except in ostial left main disease (Class IIa, level of evidence B-R). The European guidelines are similar, stating that in cases of stent failure, the use of IVUS or OCT is reasonable (Class IIa, level of evidence C-LD) [23].

IVUS was the first available intravascular imaging modality and, over the past 20 years, has demonstrated its ability to help reduce future ischemic events [24,25]. IVUS allows for vessel size and stent underexpansion identification by defining the external elastic lamina behind the stent struts, even in the presence of lipid plaque [21]. This clear visualization of the external wall layers helps optimize vessel sizing, which ultimately leads to larger-sized stent implantation and, therefore, improves post-procedural outcomes [26]. Furthermore, IVUS may also contribute to recognizing the true extent of neointimal hyperplasia [25].

Because of its higher resolution, OCT provides more detailed plaque information, including tissue characterization, lumen/neointimal interface delineation, and stent strut distribution, especially in irregularly calcified lesions. However, it has more limited depth when compared to IVUS [17,23,26]. The difference in the pathophysiology of DES vs. BMS results in different tissue characterizations in OCT. As previously mentioned, BMS-ISR typically exhibits a homogenous signal, whereas the echogenicity in DES-ISR is more heterogeneous or layered [2,27]. OCT has been extremely valuable in characterizing neoatherosclerosis, the most common finding among patients with very late stent thrombosis (ST). OCT’s ability to characterize calcification or neointimal hyperplasia may be promising in the prediction of recurrent ISR [28,29]. Indeed, OCT is more precise than angiography or IVUS in identifying morphological details such as stent malapposition, tissue prolapse/protrusion, and residual dissection, although many of them may have a benign course [20].

## 3. Invasive Physiology

In addition to intracoronary imaging, invasive physiological measurements play a significant role in guiding revascularization decisions for intermediate coronary lesions [30]. The use of fractional flow reserve (FFR) by measuring the pressure difference across a stenotic lesion during maximal hyperemia helps identify the severity of the stenosis and its impact on blood flow. In similar cases of native coronary artery disease, when the angiography severity of ISR is uncertain, angiographic physiologic guidance using methods such as fractional flow reserve (FFR) can assist in decision-making, despite the absence of randomized clinical trials in this category [2,31]. Furthermore, deferring revascularization based on a cutoff of fractional flow reserve (FFR) ≥ 0.80 or instantaneous wave-free ratio (iFR) > 0.89 has been associated with a lower rate of MACEs and a favorable prognosis [32,33]. Notably, in cases of moderate lesions, the FFR cutoff value of 0.75 for deferring angioplasty has demonstrated excellent outcomes [29,30,31,32,33,34]. Also, the research shows that FFR has a strong ability to predict major adverse cardiovascular events (MACEs) in intermediate lesions [34].

## 4. Management Strategy

**Figure 8:**
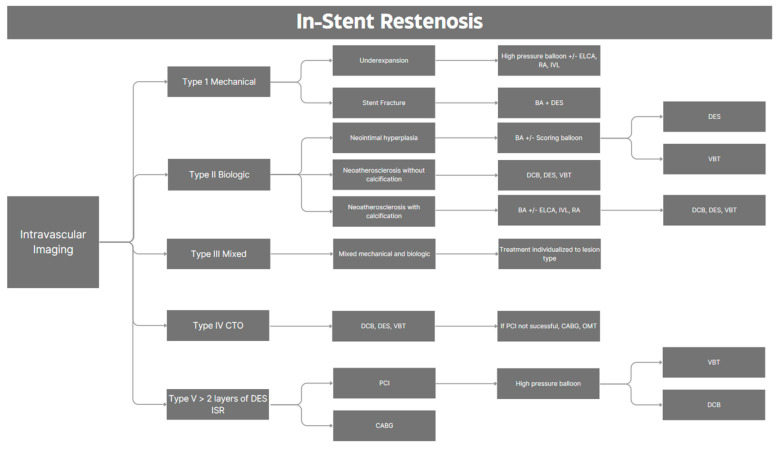
Proposed treatment algorithm for in-stent restenosis. BA: balloon angioplasty; CABG: coronary artery bypass graft; CTO: chronic total occlusion; DCB: drug-coated balloon; DES: drug-eluting stent; ELCA: excimer laser coronary atherectomy; ISR: in-stent restenosis; IVL: intravascular lithotripsy; IVUS: intravascular ultrasound; OCT: optical coherence tomography; OMT: optimal medical therapy; PCI: percutaneous coronary intervention; RA: rotational atherectomy; VBT: vascular brachytherapy.

The treatment approach for ISR is challenging due to the disease’s heterogeneous nature and a recurrence rate of 20% [4]. Various registries and clinical trials have focused on specific diagnostic and treatment methods [3,12,17,35,36,37,38,39,40,41]. The general principles for the treatment of ISR have no significant differences from those for the treatment of native coronary stenosis. Nevertheless, the presence of an existing stent frame brings some additional concerns, and issues linked to the original stent failure may need to be identified and treated accordingly in order to prevent a recurrence. In intermediate ISR lesions, it is important to determine the significance through functional tests, such as hyperemic or non-hyperemic methods or intravascular imaging modalities, such as IVUS or OCT. Different algorithms have been developed based on intravascular imaging findings to guide treatment decisions. Recently, a classification system for ISR based on intravascular imaging has been promising for enhancing outcomes [2,42]. The Waksman ISR Classification distinguishes between mechanical (Type I), biologic (Type II), or mixed (Type III) causes, including chronic total occlusions (Type IV) and lesions of DES-ISR previously treated with more than two stents (Type V) [42]. Using this tool, the mechanism of ISR should be initially identified using intravascular imaging, which differentiates between stent underexpansion (mechanical) and neointimal hyperplasia (NIH) or neoatherosclerosis (biologic). Fibrotic or heavily calcified lesions on intravascular imaging indicate lesions requiring modification to facilitate stent delivery and expansion [43]. Shlofmitz et al. [12], inspired by Waksman ISR Classification [42], proposed a treatment algorithm based on the mechanism of ISR for managing DES-ISR, as depicted in Figure 8.

Adequate lesion preparation is mandatory when treating ISR, irrespective of the final proposed treatment modality. Historically, ISR balloon angioplasty achieved adequate luminal gain through tissue compression and previous stent expansion but fell short due to recoil and tissue re-protrusion into the lumen soon after treatment [44]. Randomized trial data supporting the use of drug-coated balloons (DCBs) in angioplasty are restricted to in-stent restenosis treatment [22]. The use of DCBs to treat ISR has been given a Class I indication according to the 2018 ESC/EACTS Guidelines. DCBs have been demonstrated to be superior to plain angioplasty in BMS stents and comparable to new-generation DES [45]. Real-world data found no significant difference between DCB and thin-DES in terms of target lesion revascularization, target vessel revascularization, MI, and device-oriented composite endpoint observed during 3 years of follow-up [46]. Furthermore, Kheifets et al. showed similar outcomes between both groups after adjustment for confounding variables, although in this study, patients treated with DCB for ISR comprised a group with a higher baseline risk [47]. Recently, the FDA approved the use of DCB (paclitaxel-coated balloon) to treat coronary ISR in the USA [48]. Overall, DCBs seem to be comparable to newer-generation DES for any type of ISR [39]. However, substantial uncertainty remains on this topic, with no clear evidence currently available to guide the selection of DES types or determine whether a change in stent type is necessary for treating ISR. Further research is needed to address these gaps.

The development of DES aimed to decrease neointimal growth; however, it provides a lifelong inflammatory stimulus. Based on the 2021 ACC/AHA/SCAI Guidelines for Coronary Artery Revascularization, repeated DES has a Class I, level of evidence A indication for the treatment of ISR regarding a lower rate of restenosis and appears to be the most promising approach. Moreover, among different stent types, everolimus-eluting stents seem to be the most efficient [23].

Scoring balloons (SBs) and cutting balloons (CBs), with their special designs, can offer great performance in special settings like ISR. SBs by creating micro-incisions, or “scores”, and CBs by physically cutting the lesion in the atherosclerotic or fibrotic plaque, facilitating the maximum extrusion of neointimal tissue, which translates into a higher acute surface area and lower lumen loss in follow-up [49]. Theoretically, it may result in higher luminal diameters at lower pressure with a reduced chance of recoil. Moreover, the ISAR-DESIRE 4 trial showed that using an SB with DCB for neointimal modification lowers rates of in-segment stenosis and luminal loss compared with BA plus DCB and enhances the efficacy of DCB in ISR [50].

The introduction of dual-layer high-pressure balloons has provided a means to exert substantial pressure to tackle ISR lesions. Although high-pressure balloons are promising due to an acute luminal gain, selecting the most appropriate lesions to benefit from this approach remains a challenge [51].

Other effective modalities for debulking and modifying calcified lesions include intravascular lithotripsy (IVL) and ablative therapy such as rotational atherectomy (RA), excimer laser coronary atherectomy (ELCA), and orbital atherectomy (OA) [51]. All of these therapies play a role in the management of undilatable ISR lesions when other conventional strategies have failed, particularly when managing calcified neoatherosclerotic ISR [17].

Intravascular lithotripsy (IVL) modifies calcium through localized pressure effects on both the deep and superficial layers. Extensive analysis has demonstrated IVL’s efficacy and safety for underexpanded stent management [52]. The coronary IVL balloon catheter measures 12 mm in length, with sizes ranging from 2.5 mm to 4 mm, and delivers 80 pulses per catheter [53]. IVL may be applied in patients with calcified ISR resistant to the conventional strategy of NIH [17]. Its use is appropriate for bifurcation lesions with the possibility of wiring branches and for the treatment of lesions with circumferential (>270°) and deep calcium [54].

ELCA, introduced for over two decades, generates heat and shock waves to disrupt and modify plaque [41,55]. Currently, ELCA is considered an attractive treatment option for certain challenging subsets of lesions, such as calcified, undilatable lesions or diffuse-type ISRs [2,56]. The latter is attributed to inadequate stent expansion due to the buffer effect of neointima [56,57]. In the setting of ISR, ELCA with ablation of in-stent NIH has been associated with a higher rate of procedural success, a lower rate of complications, and improved long-term outcomes when compared with balloon angioplasty alone [57,58].

OA is a safe and effective treatment that uses a differential approach to reduce calcified plaque volumes [59,60]. OA with a dedicated wire is more suitable for de novo, complex, balloon uncrossable lesions and was approved by the US Food and Drug Administration (FDA) in 2013 for the treatment of de novo severe calcified coronary stenosis [59]. Based on the 2021 ACC/AHA/SCAI Guidelines for Coronary Artery Revascularization, the use of OA for plaque modification on fibrotic or heavily calcified lesions has a Class IIb recommendation [23].

Rotational atherectomy (RA) is beneficial in calcified neoatherosclerosis or underexpanded stents, which are resistant to balloon angioplasty. RA, with the use of diamond-coated burrs, physically removes atherosclerotic plaques [61]. In the setting of diffuse ISR, it can ablate the NIH tissue to assist stent expansion; however, its clinical significance is uncertain. Severe complications such as burr entrapment or perforation are rare in the setting of heavily calcified lesions [62]. Thus, RA can be considered a facilitative technique for completing PCI of complex, heavily calcified lesions [51].

Intravascular brachytherapy (VBT) refers to the delivery of localized radiation within the stent. It inhibits neointimal formation within the stent by delivering localized radioactive beta-radiation via a hydraulic mechanism with a Beta-Cath divide to suppress fibroblast proliferation [63]. VBT was initially used to treat BMS-ISR when randomized clinical trials demonstrated that this technique was superior to the mechanical alternatives available at the time. With DES incoming, they quickly replaced VBT, both due to the greater facility and superior results in the setting of BMS-ISR. Nowadays, VBT is mainly used to treat refractory or two-layer DES-ISR. Treatment with VBT can be repeated whenever necessary, with a 12-month interval between usages. After treatment with this therapy, patients should be maintained on lifelong antiplatelet therapy because of delayed endothelization [2,40].

### 4.1. Approach to Severely Calcified Lesions—ISR

Calcifications increase procedural complications and impair the long-term prognosis [64]. Traditionally, angiography was used to determine the extent and pattern of calcifications [65]. Despite its excellent specificity for calcium detection (98.7%), angiography is limited by its spatial resolution, which led to the integration of alternative modalities for addressing these lesions [65,66,67]. Intravascular imaging is valuable in guiding management due to the precise coronary calcium assessment provided by OCT and IVUS. However, their different characteristics define variations in the quality of information obtained from OCT or IVUS. Notably, IVUS cannot penetrate through calcium plaque, a phenomenon referred to as acoustic shadowing, which impedes the accurate assessment of calcium thickness [68]. On the other hand, the OCT light beam can penetrate calcium in depth with diminished reflection, depicting precisely the form, extent, and thickness of calcium [15]. Moreover, IVUS faces challenges in visualizing calcified deposits of small size or when they are hidden beneath large necrotic cores, making it less favorable as an intravascular imaging modality for calcified lesions [67]. Additionally, reflecting ultrasound by dense fibrotic tissue may resemble calcium in IVUS. In comparison with semi-quantitative measures of calcified plaques using IVUS, which has limitations in detecting calcium depth and microcalcifications, OCT can quantify plaque features through variables such as arc, length, thickness, area, and volume [55]. Fujino et al. devised and validated an OCT scoring system tailored for superficial calcification to find lesions that would benefit the most from modification modalities [69].

Lesions with calcium deposition exceeding a maximum angle >180°, maximum thickness >0.5 mm, and length >5 mm, as observed in OCT, comprise higher risk groups for underexpansion [67,69]. Moreover, while OCT might not be feasible in certain situations, such as moderate to severe renal failure, it can provide much more information about calcified lesions compared to IVUS and is the preferred modality in calcified lesions [53]. In fact, the differentiation of details regarding calcification as the contributing factor of stent failure—whether due to neoatherosclerosis with fibrocalcific plaque or stent underexpansion due to calcific nodules or deep circumferential calcium—is of paramount importance and can be effectively achieved through OCT. Variables such as the amount and characteristics of calcium present behind stents, calcification quantification by OCT/IVUS, and the possibility for balloon crossing and dilatation play a pivotal role in guiding the selection of the treatment options for ISR lesions [53].

Calcium debulking is mandatory for ISR in the setting of severe calcification and stent underexpansion. RA, OA, ELCA, and IVL can all be used to modify calcium plaque and facilitate stent expansion in this setting. OCT-guided protocols for atherectomy treatments, when compared to the current standard of care, could potentially emerge as an option for optimizing the management of calcified lesions.

### 4.2. Excimer Laser Coronary Atherectomy (ELCA)

The characteristics of lasers form the basis for therapeutic applications. The mechanism of laser–tissue interactions may include thermal, photoablation, photochemical, and/or photo-disruptive effects [41,70]. These effects have been utilized for intravascular treatment. The excimer laser is a pulsed gas laser that generates short-wavelength and high-energy ultraviolet pulses [51,70]. In clinical settings, excimer lasers can be safely used due to their “cold laser” property, which enables precise tissue ablation without excessive heat generation or tissue damage due to their limited penetrance [7]. A major advantage of laser catheters is their compatibility with any standard 0.014-inch guidewire. ELCA catheters are designed in four diameters: 0.9, 1.4, 1.7, and 2.0 mm for the treatment of coronary artery disease (CAD). The choice of catheter size depends on the lesion’s severity and vessel diameter [43]. The smallest is the safest catheter, while the larger provides maximum tissue ablation capabilities [71]. The laser catheter comprises two “concentric and eccentric” types of catheters based on the arrangement of laser fibers on the catheter tip. For eccentric lesions such as in-stent restenosis (ISR) and bifurcation lesions, eccentric catheters are recommended but are rarely used in current coronary intervention [41,72].

To ensure adequate ablation, the laser pulses should be advanced and retracted slowly [43]. The saline-infusion technique allows the laser to enter the tissue from the tip of the catheter, reducing the chance of dissection. In the blood-infusion technique, blood protein absorbs the majority of the delivered energy and creates microbubbles, which intensifies the risk of traumatic dissection [43,53,73]. ELCA with contrast technique can effectively disrupt intracoronary calcifications [45]. The ELEMENT registry demonstrated the safety of ELCA with contrast [71,73,74]; however, dissection remains a concerning issue. Given the incidence of significant dissection of 7% in saline-treated patients compared with 24% in blood-infusion techniques (*p* < 0.05), Deckelbaum et al. proposed integrating the saline technique in the ELCA angioplasty procedure [75].

ELCA serves as an important adjunctive for managing complicated cases, including various types of coronary artery lesions such as ACS, ISR, chronic total occlusions (CTO), non-crossable or undilatable lesions, and saphenous vein grafts [72,73]. The ULTRAMAN registry showed the safety and effectiveness of ELCA treatments, mainly for ACS and ISR, in Japan [72].

Also, ISR treatment using ELCA guided by OCT is a safe and feasible method with high success rates [76], effectively diminishing some predictors of restenosis, such as stent underexpansion due to the small final luminal surface [77]. In calcified lesions, the effectiveness of ELCA decreases, and RA serves as a cornerstone for the treatment of heavily calcified lesions [77].

Overall, from 1992 to 2018, clinical-procedural ELCA success rates for ISR ranged from 33% to 100% with a median of 91%, which improved over time [43] in higher volume centers with advanced techniques [72].

ELCA has some inherent drawbacks. Using significant amounts of contrast agents has a potential risk of renal complications. Furthermore, ELCA has a poor ablation effect on poorly visualized/heavily calcified plaques, limiting the use of laser therapy in calcified vascular disease. A combination of ELCA and RA may be an effective way of treating severe calcification. The RASER technique, which combines ELCA with RA, is applied in complicated CTO after ISR and can be explained as providing an upstream channel by ELCA to permit Microcatheter and Rota-Wire passage, while RA could fully debulk the lesion.

## 5. Conclusions

ISR remains a significant and challenging issue in the contemporary DES era, with rates continuing to increase at a range of 1% to 2% per year. Imaging techniques serve to reveal the heterogeneous nature of ISR and provide guidance for advancing management plans. Despite these challenges, there have been substantial advancements in tools and techniques to improve outcomes.

Lesion stratification, according to the Waksman ISR Classification, can guide treatment tailored to specific lesion characteristics. In specific subsets of lesions, particularly severely calcified lesions, the use of OCT as the preferred intravascular imaging technique, along with adjuvant calcium debulking therapy like ELCA, RA, OA, or IVL, should be considered. This approach aims to address the unique challenges posed by calcified lesions and optimize treatment outcomes.

A heart-team approach is strongly recommended for patients with recurrent ISR. This collaborative approach involves various medical professionals to collectively make informed treatment decisions. Furthermore, there is a clear need for more information to enhance the interventional approach for treating ISR effectively, and further research is needed to expand the interventional armamentarium to treat ISR.

## Data Availability

Not applicable.
